# Recruitment of APPL1 to ubiquitin-rich aggresomes in response to proteasomal impairment

**DOI:** 10.1016/j.yexcr.2011.02.002

**Published:** 2011-05-01

**Authors:** Iwona Pilecka, Lukasz Sadowski, Yannis Kalaidzidis, Marta Miaczynska

**Affiliations:** a International Institute of Molecular and Cell Biology, 4 Ks. Trojdena Street, 02-109 Warsaw, Poland; b Max Planck Institute of Molecular Cell Biology and Genetics, Pfotenhauerstr. 108, 01307 Dresden, Germany

**Keywords:** APPL1, adaptor protein containing PH domain, PTB domain and leucine zipper motif, EEA1, early endosome antigen 1, GFP, green fluorescent protein, HA, hemagglutinin, MIP, maximal intensity projection, APPL1, Aggresome, Endosome, Proteasome, Ubiquitination

## Abstract

Inhibitors of proteasomes have been shown to affect endocytosis of multiple membrane receptors, in particular at the step of cargo sorting for lysosomal degradation. Here we demonstrate that the inhibition of proteasomes causes specific redistribution of an endosomal adaptor APPL1, which undergoes initial solubilization from APPL endosomes followed by clustering in the perinuclear region. MG132 treatment decreases APPL1 labeling of endosomes while the staining of the canonical early endosomes with EEA1 remains unaffected. Upon prolonged treatment with proteasome inhibitors, endogenous APPL1 localizes to the site of aggresome formation, with perinuclear APPL1 clusters encapsulated within a vimentin cage and co-localizing with aggregates positive for ubiquitin. The clustering of APPL1 is concomitant with increased ubiquitination and decreased solubility of this protein. We determined that the ubiquitin ligase Nedd4 enhances polyubiquitination of APPL1, and the ubiquitin molecules attached to APPL1 are linked through lysine-63. Taken together, these results add APPL1 to only a handful of endogenous cellular proteins known to be recruited to aggresomes induced by proteasomal stress. Moreover, our studies suggest that the proteasome inhibitors that are already in clinical use affect the localization, ubiquitination and solubility of APPL1.

## Introduction

Receptor-mediated endocytosis is a process by which cells internalize extracellular ligands. It is initiated by the inward budding of plasma membrane vesicles containing ligands bound to specific transmembrane receptors. Among other routes, receptors can be internalized via clathrin-coated pits that pinch off, lose their clathrin coat and fuse with an endosomal compartment where the cargo is sorted towards recycling or degradation in lysosomes. Several endosomal populations can be distinguished based on the presence of specific markers as well as functional and morphological characteristics. Early endosomes that contain the small GTPase Rab5 and EEA1 (early endosome antigen 1) are the first cargo sorting platform [1]. A subpopulation of early endosomes, initially characterized by the presence of APPL1 protein as a unique marker [2], are called APPL1-positive vesicles or APPL endosomes [3]. From early endosomes various types of cargo are transported to recycling endosomes, late endosomes and lysosomes, or towards the Golgi apparatus.

Ubiquitination acts as a signal for the internalization and sorting of plasma membrane proteins along the endocytic route (reviewed in [4]). In contrast to the well-established function of polyubiquitination in proteasome-dependent protein degradation, the ubiquitination involved in endocytosis does not lead to protein destruction in proteasomes. Still, during endocytic trafficking of epidermal growth factor (EGF) receptor there are several stages sensitive to the inhibitors of proteasome, namely the sorting from early to late endosomes and the translocation of activated EGF receptor from the outer limiting membrane to the inner membranes of multivesicular bodies (MVBs) [5,6]. Ubiquitin-dependent sorting is specific to endocytosed cargo, as proteasomal inhibitors block ligand-induced internalization of glutamate receptors, but not of transferrin receptor [7]. In general, inhibition of proteasomes blocks the sorting of membrane receptors towards degradation without interfering with the transport of soluble proteins or recycling cargo [8]. The application of proteasome inhibitors decreases the rates of lysosomal degradation of multiple transmembrane receptors, including those for EGF [9], growth hormone and nerve growth factor [8], platelet-derived growth factor [10], hepatocyte growth factor [11], interleukin-2 [12] and low-density lipoprotein receptor-related protein [7].

In contrast to the general knowledge about the involvement of ubiquitin–proteasome system in endocytosis, specific effects of proteasome inhibitors on early endosomes are not known. In particular, we were interested in the fate of the subpopulation of early endosomes marked by the presence of APPL1, which act as a sorting and signaling platform. APPL endosomes are positive for Rab5 but not EEA1 (which is a marker for the canonical early endosomes), and are preferentially localized underneath the plasma membrane [2]. APPL1 is primarily recruited to endocytic membranes through binding to the small GTPase Rab5. The endocytic compartments are highly dynamic, and changes in the composition of phosphoinositides on early endocytic membranes cause a selective recruitment of either APPL1 or EEA1 to Rab5-positive endosomal vesicles [3]. APPL endosomes participate in endocytic trafficking of EGF receptor [2] and transferrin receptor (our unpublished results). In addition, they function as platforms for the assembly of signaling complexes that control distinct signal transduction pathways, e.g. the Akt and MAPK pathways [3,13]. APPL1, a unique marker of APPL endosomes, interacts with Akt and phosphatidylinositol 3-kinase, as well as with several membrane receptors (including adiponectin receptor 1 and 2, netrin-1 receptor DCC, follicle stimulating hormone receptor**,** and nerve growth factor receptor TrkA), contributing to signal transduction downstream of these receptors (reviewed in [14]). The cellular functions of APPL1 are not limited to endocytosis, and include also regulation of gene transcription, metabolism, cell proliferation and cell survival [14]. Recently, we reported that APPL1 can activate β-catenin-dependent transcription [15] and interact with the NuRD chromatin remodeling and histone deacetylase complex [2,16].

While proteasomal inhibition affects endocytic trafficking already upon short application (1 h or less), prolonged (12–24 h) treatment causes massive changes in the turnover of cellular proteins. Proteasome deficiency stimulates a cellular stress response by inducing time- and dose-dependent inhibition of cell growth, arrest of the cell cycle, apoptosis, as well as the loss of mitochondrial membrane potential and increase in the intracellular ROS levels [17]. Compromised proteasomal function leads to the accumulation of misfolded proteins which become sequestered into large, insoluble, non-membranous protein deposits called aggresomes. These structures were first described by Wojcik et al. in HeLa cells treated with a peptide aldehyde proteasomal inhibitor PSI [18], and named aggresomes by Johnston et al. [19]. Aggresomes appear either as a single sphere with a diameter of 1–3 μm positioned at an indentation of the nucleus, or as an extended ribbon around the nuclear envelope [20]. Aggresomes assemble in the perinuclear region near the microtubule organizing center, and their formation involves trafficking of aggregated proteins along microtubules and reorganization of intermediate filaments [19–21]. Aggresomes recruit chaperones, ubiquitination enzymes and components of the ubiquitin–proteasome machinery, to help in the disposal of aggregated proteins. The process of aggresome clearance is largely dependent on the autophagy–lysosome system. The cellular site of aggresome formation is enriched in double-membrane vesicles representing autophagosomes, and blockage of autophagy impairs the clearance of aggresomes [22]. Lysosomes gather close to the aggresome and eventually digest the proteins forming the inclusions.

Here, we aimed to characterize an effect of proteasomal inhibition on APPL endosomes. We analyzed the localization and morphometric features of APPL1-positive endosomes upon a range of incubation times with proteasome inhibitors. Strikingly, we observed initial solubilization of APPL1 from endosomal membranes followed by its relocalization to aggresomes which correlated with increased ubiquitination and insolubility of APPL1.

## Materials and methods

### Antibodies and chemicals

Anti-APPL1 polyclonal antibodies against C-terminal peptides were raised in rabbits (Eurogentech) and previously described [2,15]. The following mouse monoclonal antibodies were used for immunofluorescence: anti-EEA1 and GM130 (BD Transduction Laboratories), CD63 (Developmental Studies Hybridoma Bank), Rab5 (D-11, Santa Cruz Biotechnology), ubiquitin (FK1, Biomol), vimentin and GFP (Sigma-Aldrich). Alexa Fluor 405-, 488- and 555-conjugated anti-mouse and anti-rabbit antibodies, as well as EGF and transferrin labeled with Alexa Fluor 488 were from Invitrogen. Additional antibodies were used in Western blotting: mouse antibodies against HA and ubiquitin P4D1 (Santa Cruz Biotechnology), α-tubulin, β-actin and FLAG (M2) (Sigma-Aldrich), HDAC2 (Upstate), and goat antibody against GFP (MPI, Dresden). Secondary horseradish peroxidase-conjugated antibodies were from Jackson ImmunoResearch. MitoTracker Orange probe (Invitrogen) was used according to provider's instructions. Cycloheximide, 4′,6-diamidino-2-phenylindole (DAPI), N-ethylmaleimide (NEM) and nocodazole were purchased from Sigma-Aldrich. Proteasome inhibitors MG132, ALLN and clasto-lactacystin β-lactone were from Sigma-Aldrich, and bortezomib from LC Laboratories. The inhibitors were diluted in dimethyl sulfoxide (DMSO) and an equivalent volume of DMSO was used in all experiments as a solvent control.

### Plasmids and siRNA reagents

The constructs of untagged, Myc-tagged and GFP-tagged APPL1 were previously described [2]. The expression constructs of FLAG- and HA-tagged ubiquitin wild type, HA-tagged ubiquitin mutant K63R, HA-tagged c-Cbl and untagged Nedd4 were a gift from Ivan Dikic. The HA-ubiquitin K63-only expression construct was provided by Ted M. Dawson (Addgene plasmid 17606) [23]. The mouse HA-Nedd4-1 expression construct was provided by Allan M. Weissman (Addgene plasmid 11426) [24]. The mouse GFP-Eps15 construct was previously described [25].

We used two double stranded siRNA oligonucleotides against human APPL1 from HP GenomeWide siRNA collection (Qiagen): (APPL1-a) Cat. No. SI02652125, (APPL1-b) Cat. No. SI03128979. Alongside, two siRNA negative controls were used (Qiagen): (control-a) Cat. No. 1027280 and (control-b) Cat. No. 1022076.

### Cell culture and transfections

Human cell lines HEK293 (embryonic kidney) and HeLa (cervix carcinoma) were cultured as previously described [15]. HEK293 cells were transiently transfected using the calcium phosphate method [26]. For microscopical analysis, HeLa cells were transfected with 0.5 μg of plasmid DNA in 24-well plates using FuGene reagent (Roche), treated as indicated and fixed 48 h post-transfection. Duplexes of siRNA (10 nM) were delivered to HeLa cells using HiPerFect reagent (Qiagen) according to manufacturer's instructions.

### Immunofluorescence

HeLa cells were processed for immunofluorescence staining as previously described [16]. Images were acquired on a Zeiss LSM510 laser scanning confocal microscope with a 63×/1.4 Plan-Apochromat oil immersion objective and argon and HeNe lasers. Z-stacks were built and converted to maximal intensity projections (MIPs) using ZEN 2009 Light Edition. The presented microscopy images were assembled using Adobe Photoshop 7.0.

### Quantification of microscopical images

For morphometric image analysis we used the custom-designed image analysis software MotionTracking/Kalaimoscope (www.kalaimoscope.com) [27,28]. In brief, endosomes were automatically identified as fluorescent objects based on the user-defined parameters i.e. pixel fluorescence intensity, minimum area and resolution limit, using an algorithm implemented in MotionTracking. The software created a synthetic image with identified vesicles and subtracted extracellular and intracellular background, and performed statistical analysis of modelled endosomes. Statistical parameters were calculated per overall area occupied by the cells. Quantification of the endocytic phenotypes included the following parameters: the number of vesicles, the mean apparent area of the cross section of vesicles expressed in μm^2^ (corresponding to the average vesicle size), and the total fluorescence of a fluorophore detected in all vesicles, defined as the total integral vesicle intensity. These parameters were calculated separately for red (APPL1) and green (EEA1) channels based on more than a hundred of imaged cells per condition.

For quantification of APPL1 clusters, we counted the HeLa cells containing perinuclear clusters positive for APPL1. For quantification of ubiquitin aggregates, we counted the HeLa cells containing four arbitrary chosen and easily distinguishable types of ubiquitin distribution. The calculations were based on the indicated number of scored cells and expressed as a percentage of all counted cells. Images from confocal microscope were taken with a maximally opened pinhole (8.89 Airy Units, corresponding to 6.9-μm-thick section) in order to view the entire cell volume.

### Lysis, Western blotting and immunoprecipitation

Cells were lysed in RIPA buffer containing 1% Triton X-100, 0.5% deoxycholate, 0.1% SDS, 50 mM Tris–Cl (pH 7.4), 150 mM NaCl, 0.5 mM EDTA and protease inhibitor cocktail. Soluble lysate samples of 10–20 μg total protein were subjected to SDS-PAGE. Protein concentration was measured with Bradford assay (Roth). Where appropriate, insoluble fractions were prepared from cellular pellets formed upon extraction for 20 min on ice with RIPA buffer, followed by centrifugation at 20,000*g* for 15 min. The pellets were washed and boiled in SDS-PAGE sample buffer. Total cellular lysates were prepared by adding hot SDS-PAGE sample buffer to the dish with adherent cells, scraping and boiling prior to loading on the gel. Resolved proteins were transferred to nitrocellulose membrane (Whatman), probed with specific antibodies, and detected with enhanced chemiluminescence.

Proteins of interest were immunoprecipitated from soluble lysates containing 100–200 μg of protein by 2-h incubation with an appropriate antibody at 4 °C with constant rotation, recovery of immunocomplexes on protein A sepharose beads (Roche), washing in IP buffer (1% Triton X-100, 50 mM Hepes (pH 7.4), 150 mM NaCl, 1 mM EDTA, 1 mM EGTA, 10% glycerol), and elution with SDS-PAGE sample buffer. Where indicated, non-immune rabbit immunoglobulins were used in control immunoprecipitations.

### Statistical analysis

The statistical significance was assessed by using the Mann–Whitney *U* test. The level of significance was set at *p* < 0.05. Analyses were performed using GraphPad Prism software.

## Results

### Inhibition of proteasomes causes redistribution of APPL1

**Fig. S1:**
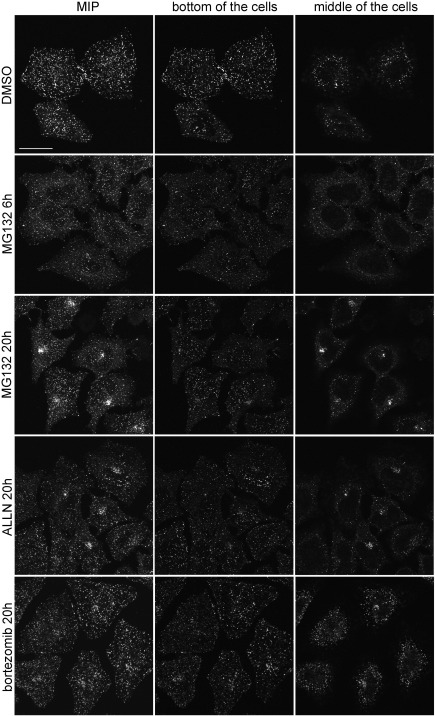
Changed distribution of APPL1 upon proteasomal inhibition. HeLa cells seeded on coverslips were treated with 5 μM MG132, 50 μM ALLN, 1 μM bortezomib, DMSO (the solvent control). After indicated times, the cells were fixed, permeabilized, and stained for APPL1 followed by Alexa555-conjugated anti-rabbit antibodies. The left panels show maximum intensity projections (MIP) of z-stacks containing 7–9 confocal images taken every 0.5 μm throughout the entire volume of the cells. The middle panels show individual confocal scans taken at the bottom of the cells, while the right panels show scans from the middle of the same cells. Bar, 20 μm.

**Fig. 1:**
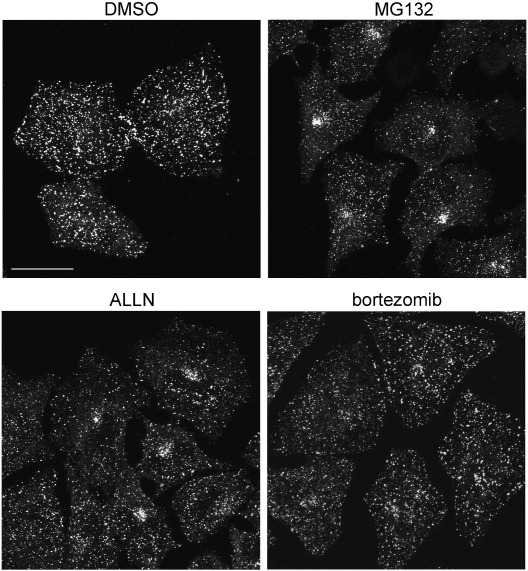
Changed distribution of APPL1 upon proteasomal inhibition. HeLa cells seeded on coverslips were treated for 20 h with 5 μM MG132, 50 μM ALLN, 1 μM bortezomib, or a corresponding amount of DMSO (the solvent control). The cells were fixed, permeabilized, and stained for APPL1 followed by Alexa555-conjugated anti-rabbit antibodies. The panels show maximum intensity projections (MIP) of z-stacks containing 7–9 confocal images taken every 0.5 μm throughout the entire volume of the cells. The examples of single scans used for the construction of MIPs are shown in Fig. S1. Bar, 20 μm.

In order to investigate the effect of proteasome inhibitors on APPL endosomes, we looked at the distribution of endogenous APPL1 in HeLa cells treated with MG132, ALLN, or bortezomib. Both MG132 and ALLN are cell-permeable peptide aldehydes commonly used as reversible inhibitors of the chymotrypsin-like proteolytic activity of the proteasome. Bortezomib (Velcade), a dipeptide boronic acid analogue, has increased affinity for the catalytic subunits of the proteasome and reduced multidrug resistance sensitivity. It represents a prototype of highly effective anti-cancer agents, used for treatment of two B-cell malignancies: multiple myeloma and mantle cell lymphoma [29,30]. We used MG132 at two concentrations (5 or 10 μM), ALLN at 50 μM, and bortezomib at 1 μM, for times ranging from 1 h till 20 h. Fig. 1 shows representative images of maximal intensity projection (MIP) covering the entire cell volume. The individual confocal scans taken from the bottom and the middle of the cells are shown in Fig. S1. In DMSO-treated control cells, APPL1-positive endosomes are dispersed in the cytoplasm and in particular concentrated underneath the plasma membrane. One-hour treatment with the inhibitors did not cause any observable changes compared to the control cells (data not shown). Both 6- and 20-h treatments with the inhibitors caused a marked decrease in the number of APPL endosomes close to the ventral cell membrane facing the glass (Fig. S1, middle panels). Interestingly, we observed clusters of APPL1-positive structures formed in the perinuclear region upon 20-h treatment with MG132, ALLN and bortezomib (Fig. 1 and Fig. S1, right panels).

**Fig. S2:**
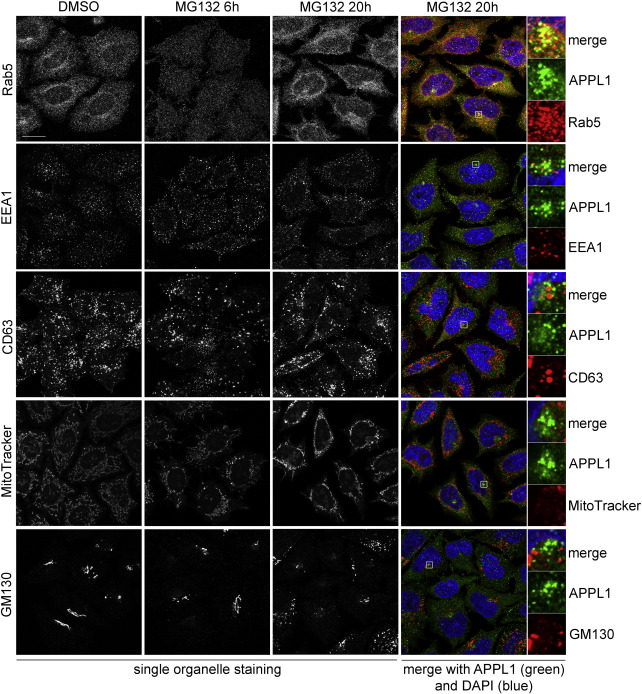
Effect of MG132 on the localization and morphology of cellular compartments. HeLa cells were treated with control DMSO for 20 h or 10 μM MG132 for 6 or 20 h, fixed, permeabilized and stained for: Rab5 or EEA1 (early endosomes), CD63 (late endosomes), MitoTracker Orange (mitochondria), and GM130 (the Golgi apparatus). All panels represent single confocal scans. Bar, 20 μm. The color panels show co-staining for the organelles (red) and APPL1 (green) from cells treated with MG132 for 20 h. Insets show magnification of APPL1 clusters; inset size: 5.3 μm.

In order to check whether the changes in APPL1 distribution upon prolonged proteasomal inhibition are specific, we looked at the localization of other proteins residing on early or late endosomes as well as at the distribution of other cellular organelles (Fig. S2). We found that the distribution of early endosomal marker Rab5, present on APPL endosomes, followed the pattern of changes displayed by APPL1: both proteins were solubilized from endosomal membranes upon 6-h MG132 treatment, and co-localized in perinuclear clusters upon 20-h treatment. In contrast, EEA1, selectively residing on the canonical early endosomes, showed no obvious redistribution (Fig. S2). CD63, a marker of late endosomal and lysosomal membranes, changed the localization in cells treated for 20 h with MG132, but it did not co-localize with APPL1 clusters (Fig. S2). The same was true for mitochondria (stained with MitoTracker Orange probe) and the Golgi apparatus (stained with anti-GM130 antibody) (Fig. S2). The observed redistribution of cellular organelles was in agreement with the literature data [31–33]. We conclude that proteasomal stress selectively affects endosomes containing APPL1 and Rab5.

**Fig. S3:**
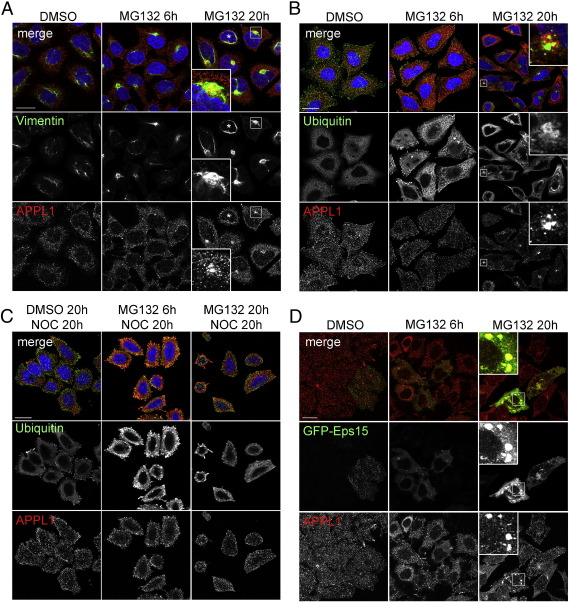
Distribution of endosomes harboring APPL1 or EEA1 in HeLa cells treated with MG132 for 6 or 20 h, or control DMSO for 20 h. Cells were fixed, permeabilized and co-stained with APPL1 and EEA1. The panels show representative series of MIP and single images taken at three confocal planes (bottom, middle, top of the cells), used for analysis in Fig. 2A-C. Bar, 20 μm.

**Fig. 2:**
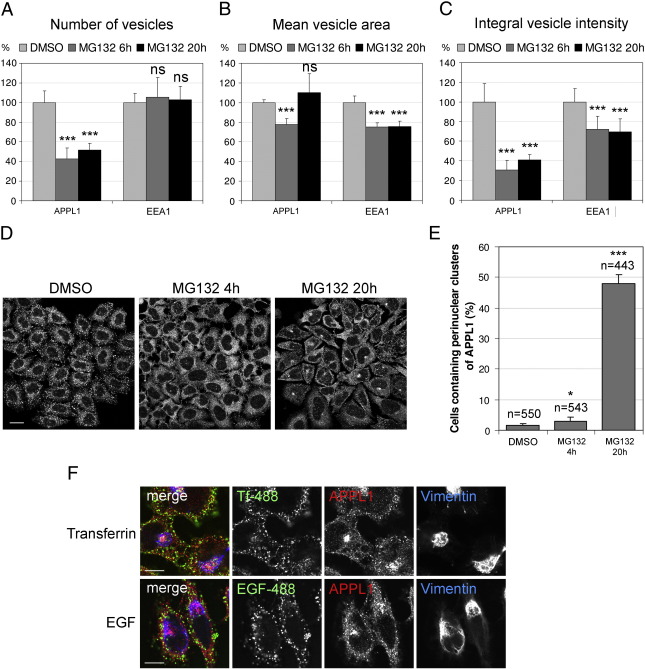
MG132 influences the properties of an endosomal subpopulation harboring APPL1. (A–C) Quantification of the distribution and morphometric properties of APPL1 and EEA1 endosomes. A detailed description is given in Material and methods section. Ten MIPs were analyzed for every condition; each MIP was assembled from images taken at three confocal planes (bottom, middle, and top of the cells). The results represent averaged values of each parameter with standard deviation, and are expressed as a percentage of control values obtained from DMSO-treated cells. (A) The number of vesicles containing APPL1 or EEA1. (B) Mean area of vesicles positive for APPL1 or EEA1. (C) Total integral fluorescence intensity of vesicles containing APPL1 or EEA1. (D) Clustering of APPL1 in HeLa cells treated with proteasome inhibitor. Cells were treated with MG132 for 4 or 20 h, or control DMSO for 20 h. The panels show representative images used for analysis in E. Bar, 20 μm. (E) Quantification of the perinuclear clusters of APPL1. Seven random images were taken for each condition and the cells containing clustered APPL1 were counted and expressed as a percentage of all scored cells (n indicates the number of scored cells). The graph shows averaged results with standard deviation of an experiment done in duplicate, representative of three independent experiments. (F) HeLa cells seeded on coverslips were treated with DMSO or 5 μM MG132 in the presence of fluorescently labeled EGF-Alexa 488 (1 μg/ml) or transferrin-Alexa 488 (2.5 μg/ml). After 18 h of continuous uptake the cells were fixed and co-stained for APPL1 and vimentin. Bar, 10 μm. A–C and E: *, *p* < 0.05; ***, *p* < 0.001; ns, not significant compared with DMSO control (Mann–Whitney test).

In order to quantify the extent of observed changes in APPL1 localization, morphometric features of APPL1- and EEA1-containing endosomes were compared using the image analysis software MotionTracking [27,28]. Upon analysis of the double-immunolabelled images, we quantified the following parameters for APPL1 and EEA1 endosomes: the number of vesicles, the average vesicle size (mean apparent area), and the total integral vesicle intensity (sum of fluorescence of all vesicles) (Fig. 2A–C and Suppl. Table 1). Fig. S3 shows representative images used for the measurements. We observed that the number of APPL endosomes decreased upon MG132 treatment (to 42% and 51% of control value after 6 and 20 h of MG132 treatment, respectively) (Fig. 2A). Proteasomal inhibition caused an initial decrease of mean area of APPL vesicles (to 78% of control after 6 h), which returned to the control levels after 20 h (Fig. 2B). In addition, the overall number of APPL1 molecules residing on endosomes dropped upon MG132 treatment (measured as total integral intensity of all vesicles, Fig. 2C) to 31% and 41% of control value after 6 and 20 h, respectively.

At the same time, the EEA1 endosomes displayed much less changes. The number of EEA1 endosomes was not affected by MG132 treatment (Fig. 2A). The area of EEA1 endosomes was consistently decreased by MG132 treatment at 6 and 20 h (to 75% of control; Fig. 2B). Correspondingly, the integral vesicle intensity for EEA1 was reduced to 72% and 70% of control, for 6- and 20-h MG132 treatment, respectively (Fig. 2C).

Based on this data, we could conclude the following: (a) Inhibition of proteasomes decreased the number of detectable APPL endosomes by half, while it did not affect the number of observed EEA1 vesicles; (b) Mean size of APPL vesicles initially decreased and then returned to the control levels upon MG132 treatment, with a heterogeneous vesicle population after 20 h (bigger vesicles in the perinuclear cluster and smaller ones scattered in the cytoplasm); (c) MG132 treatment solubilized APPL1 from vesicles, as the massive decrease in the amount of APPL1 bound to vesicles could not be explained by the observed changes in the vesicle size; (d) MG132 treatment reduced the size of EEA1 vesicles and, proportionally, the amount of vesicle-bound EEA1. Clearly, MG132 affects both early endosomal compartments (as expected due to multiple morphological changes seen in other membraneous cellular structures). However, the extent and timing of MG132 effects seem to be substantially different in the case of APPL1 and EEA1 endosomes. In the case of EEA1, MG132 treatment primarily affected the vesicle size. In the case of APPL1, changes were much more pronounced and involved the decreased amounts of vesicle-bound APPL1 molecules as well as the decreased number of detectable APPL1-positive vesicles.

Although the morphometric image analysis provided us with quantitative data, due to averaging of analyzed parameters within endosomal populations it was not optimal for characterizing distinct patterns of APPL1 redistribution upon the two time periods of MG132 treatment. To complement the initial analysis, we counted the number of HeLa cells containing perinuclear clusters of APPL1 upon treatment with DMSO or MG132 for 4 or 20 h. Representative images are shown in Fig. 2D. While clusters of APPL1 were a rare event in control cells or upon 4-h MG132 treatment, upon 20-h MG132 application the number of cells containing the perinuclear clusters of APPL1 reached almost 50% of all counted cells (Fig. 2E).

**Fig. 3:**
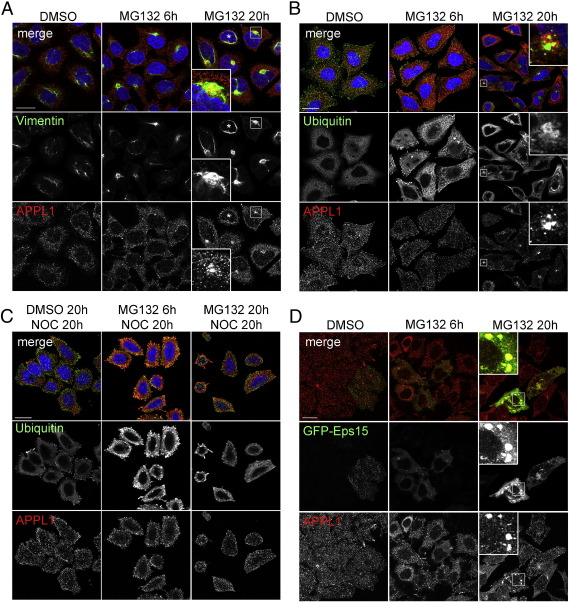
The perinuclear clusters of APPL1 co-localize with the sites of aggresome formation. (A) HeLa cells seeded on coverslips were treated with control DMSO for 20 h or 10 μM MG132 for 6 or 20 h, fixed and co-stained for endogenous vimentin and APPL1 together with the nuclear staining (blue) by DAPI. Asterisks indicate a cell without an aggresome after 20-h treatment with MG132. (B) HeLa cells were treated as in A, and stained for endogenous ubiquitin and APPL1 together with the nuclear staining (blue) by DAPI. (C) HeLa cells were treated with 5 μM nocodazole (NOC) for 20 h in parallel to the treatment as in A. Cells were stained as in B. (D) HeLa cells were transiently transfected with GFP-Eps15, after 24 h treated as in A, and stained for an endogenous APPL1. All scale bars A–D, 20 μm. Insets in A, B and D show magnification of APPL1 clusters from cells treated with MG132 for 20 h.

At this point it was important to determine whether perinuclear clustering of APPL1 represents its recruitment to endosomes relocalized in the cell center or a formation of proteinaceous aggregates without a surrounding membrane. We checked this by employing continuous uptake of fluorescently labeled cargo during the incubation with MG132 for 18 h. We used transferrin that is constitutively recycled and EGF that undergoes degradation in lysosomes, both labeled with Alexa Fluor 488. In addition, the cells were stained for APPL1 and for vimentin in order to visualize aggresomes (see Fig. 3A for more explanations). As shown in Fig. 2F, neither transferrin nor EGF accumulated at the sites of perinuclear APPL1 clusters, suggesting that these are not functional endosomes transporting extracellular cargo. In the cells treated with MG132 for 18 h some peripheral APPL1-positive endosomes contained transferrin, while there was no co-localization of APPL1 and EGF. As previously reported [6], MG132 markedly decreased degradation of EGF, which accumulated in the cells treated with MG132 to a larger extent than in control-treated cells (data not shown).

Taken together, the above-presented data allowed concluding that upon a prolonged proteasomal inhibition APPL1 is solubilized from endosomes and relocalizes to the perinuclear region where it forms clusters, which are most likely proteinaceous aggregates. These clusters are not functional cargo-bearing vesicles and do not co-localize with early or late endosomes, mitochondria or the Golgi apparatus.

### Co-localization of clustered APPL1 with aggresomes upon proteasomal inhibition

As the observed perinuclear clusters of APPL1 did not represent functional endosomes, we wished to further characterize these aggregates. We performed a series of co-stainings of APPL1 with proteins known to participate in aggresome assembly in order to check whether the perinuclear clusters of APPL1 co-localize with an aggresome.

**Fig. S4:**
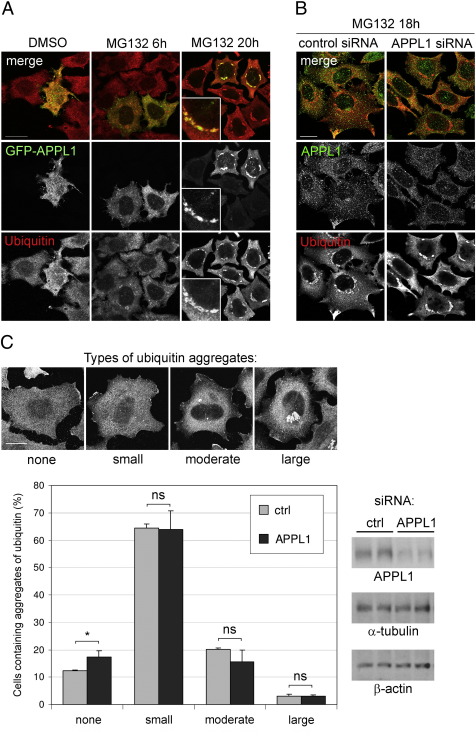
Common effects of three proteasome inhibitors on APPL1 clustering. HeLa cells seeded on coverslips were treated with 5 μM MG132, 1 μM bortezomib or 10 μM clasto-lactacystin β-lactone for 20 h. Then the cells were fixed and co-stained for endogenous vimentin and APPL1 together with the nuclear staining (blue) by DAPI.

One of the most typical components of aggresomes is an intermediate filament protein vimentin that during aggresome formation is displaced from its normal fibrillar cellular distribution and forms a cage surrounding the core of aggregated proteins [19–21]. Fig. 3A shows that APPL1 clusters are confined within the collapsed vimentin cage in cells treated for 20 h with MG132. Markedly, under this treatment every cell containing an aggresome surrounded by vimentin also contains clustered APPL1. The one cell marked with an asterisk in Fig. 3A has neither collapsed vimentin nor clustered APPL1. Similarly, we saw co-localization of APPL1 and vimentin in cells treated for 20 h with other proteasomal inhibitors, bortezomib and clasto-lactacystin β-lactone (Fig. S4). Interestingly, already upon 6 h of MG132 treatment we could observe cells containing collapsed vimentin cages, but no APPL1 clusters (Fig. 3A).

Another component of aggresomes formed in response to a failure of proteasome machinery are ubiquitinated proteins [20,21]. We checked whether clusters of APPL1 form at the sites of ubiquitin aggregation by co-staining of APPL1 and ubiquitin. Indeed, all clusters of APPL1 were localized at a close proximity to the perinuclear ubiquitin aggregates (Fig. 3B).

The third feature of aggresomes is their dependence on transport along microtubules [19]. We co-treated cells with MG132 and nocodazole, a microtubule-disrupting agent, which prevents the assembly of aggregated proteins into a large perinuclear aggresome. Upon treatment with MG132 for 20 h in the presence of nocodazole, we could neither see the formation of ubiquitin aggregates nor clustering of APPL1 (Fig. 3C). However, nocodazole did not affect the localization of APPL1 in DMSO-treated cells (Fig. 3C).

It has been previously reported that several endocytic proteins are recruited to aggresomes via their interaction with a protein called ubiquilin that bears ubiquitin-like domain (UBL). These are proteins possessing ubiquitin-interacting motifs (UIMs): Eps15, Hrs and STAM2 [34]. In particular, Eps15 has been shown to participate in the formation of aggresomes [35]. We expressed GFP-Eps15 in HeLa cells and checked its localization in parallel to an endogenous APPL1 upon treatment with DMSO or MG132 for 6 or 20 h (Fig. 3D). There was no co-localization of Eps15 and APPL1 in control cells or cells treated with MG132 for 6 h. Interestingly, upon 20-h MG132 treatment we observed not only an aggregation of both proteins in the same region of the cells, but also a recruitment of APPL1 to the GFP-positive aggregates in cells expressing high levels of GFP-Eps15 (Fig. 3D). In agreement with these observations, we could also detect Eps15 co-precipitating with APPL1 (data not shown).

On the basis of the above microscopical data, we conclude that the perinuclear clusters of APPL1 formed upon proteasomal inhibition co-localize with the commonly accepted aggresome markers.

### Role of APPL1 in the formation and clearance of aggresomes

**Fig. 4:**
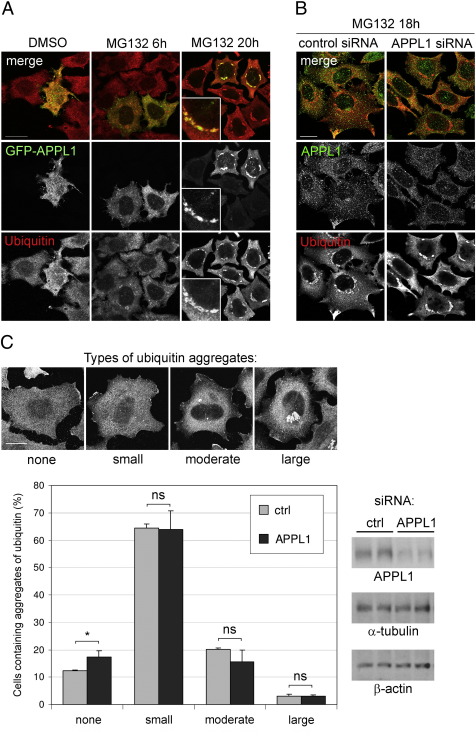
The role of APPL1 in aggresome formation and clearance. (A) HeLa cells seeded on coverslips were transiently transfected with GFP-APPL1; 24 h later they were treated with 10 μΜ MG132 for additional 6 or 20 h, then fixed and stained for GFP and endogenous ubiquitin. Insets show a magnification of aggregates of APPL1 from cells treated with MG132 for 20 h. Bar, 20 μm. (B) HeLa cells seeded on coverslips were transfected with 10 nM siRNA (either control or targeting APPL1). After 24 h cells were treated with 5 μM MG132 for 18 h, fixed and co-stained for ubiquitin and APPL1. Bar, 20 μm. (C) HeLa cells seeded on coverslips were transfected with two independent control siRNAs and two siRNA duplexes against APPL1. After 24 h cells were treated with 5 μM MG132 for 18 h, washed and allowed to grow in normal medium for additional 24 h. Cells were fixed and co-stained for ubiquitin, APPL1 and DAPI. Cells containing clusters of ubiquitin were grouped into four types based on their size (representative images are shown in upper panel; scale bar 20 μm). The cells were counted and expressed as a percentage of scored cells (total number of cells *n* = 566 for control siRNAs and *n* = 713 for APPL1 siRNAs). The graph shows averaged results obtained from two independent siRNA sequences with standard deviation. *, *p* < 0.05; ns, not significant compared with control siRNA (Mann–Whitney test). Right panels show the efficiency of APPL1 knockdown in comparison to α-tubulin and β-actin levels in cells treated with control (ctrl) and APPL1 siRNA duplexes. The cells used for Western blot were grown and treated in parallel to the cells used for microscopical analysis and they were either lysed or fixed at the same time.

Since other endocytic proteins (i.e. Eps15) have been shown to affect aggresome formation [35], we checked the influence of APPL1 on proteasome-dependent aggregation. We overexpressed GFP-APPL1 in HeLa cells and treated them with control DMSO or MG132 for 6 or 20 h. Similarly to an endogenous protein, GFP-APPL1 was localized in punctate endosomal structures under control conditions, as previously reported [2] and was solubilized from endosomes upon 6 h of MG132 treatment. Upon 20 h of MG132, GFP-APPL1 was concentrated in multiple large perinuclear structures which were encapsulated by ubiquitin (Fig. 4A). To test whether APPL1 overexpression influences formation of aggresomes, we compared the localization of ubiquitin-rich aggregates in the transfected versus untransfected cells upon treatment with MG132 for 6 and 20 h. Ubiquitin accumulated in perinuclear areas already at 6 h of MG132 treatment, and after 20 h virtually all cells contained aggregates of ubiquitinated proteins. However, the extent of their formation in cells overexpressing GFP-APPL1 did not differ from the neighboring untransfected cells, indicating that an increased level of APPL1 does not modulate or enhance the proteasome-dependent aggregation of ubiquitinated proteins (Fig. 4A). Similarly, knockdown of APPL1 by siRNA had no significant effect on the formation of MG132-indduced ubiquitin-rich aggregates (Fig. 4B). Since in HeLa cells we achieved only partial silencing of APPL1, it remains formally possible that the complete knockout would affect aggregation of ubiquitinated proteins upon proteasomal impairment. However, it is rather likely that clustering of APPL1 is an event rather successive to than causative of aggresome formation.

The aggregates formed upon treatment with MG132 are reversible, and typically cells return to their normal morphology within 24–72 h after the removal of the inhibitor. To assess the effect of APPL1 on the clearance of aggresomes, after 18-h incubation with MG132 we washed the cells and allowed them to grow for the next 24 or 48 h in a normal medium. Upon 24 h of recovery time, ubiquitin was evenly distributed throughout the cytoplasm, with small cytoplasmic aggregates in 64% of the cells (Fig. 4C). The aggregates were not visible in 12% of control cells, while the cells containing the bigger clusters were classified as “moderate” (20%) or “large” (3%) based on their size (Fig. 4C). After 48-h recovery time, all cells had none or only small ubiquitin aggregates. We reduced the level of APPL1 protein with the help of two independent siRNA duplexes, and counted the cells containing various patterns of ubiquitin distribution after 24-h recovery time. As shown in Fig. 4C, silencing of APPL1 had no substantial effects on the clearance of ubiquitin aggregates. However, compared to control cells, in the cells treated with siRNA targeting APPL1, we observed a 5% increase in the number of cells without clusters, which could argue that, if at all, APPL1 might only have a slight modulatory impact on the dynamics of aggresome clearance.

### Ubiquitination of APPL1

**Fig. 5:**
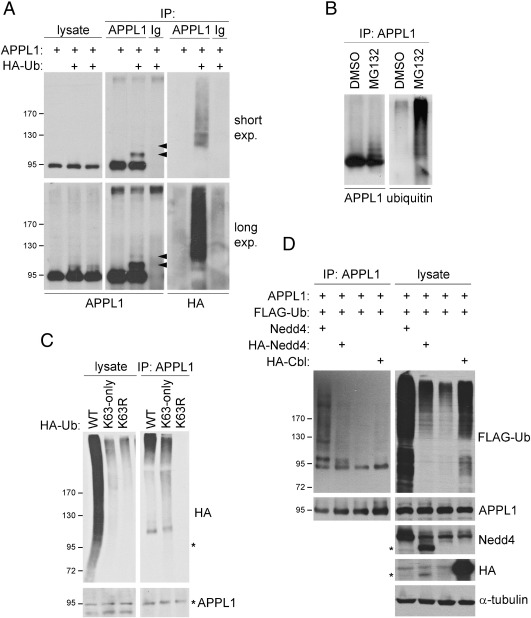
Ubiquitination of APPL1. (A) Ectopically expressed APPL1 is ubiquitinated in HEK293 cells. Cells were transiently transfected with untagged APPL1 and HA-ubiquitin (HA-Ub) as indicated, and 48 h later lysed in RIPA buffer. APPL1 and tagged ubiquitin were detected by Western blotting in the immunoprecipitates (IP) of APPL1 and control rabbit immunoglobulins (Ig). The arrowheads indicate the positions of ubiquitinated APPL1 species. Both short and long exposures of the blots are shown. (B) Ubiquitination of endogenous APPL1 in HeLa cells is enhanced by MG132. HeLa cells were treated with DMSO or 5 μM MG132 overnight and lysed in RIPA supplemented with 10 mM NEM. The resulting lysates were subjected to immunoprecipitation using anti-APPL1 antibodies. APPL1 and ubiquitin were detected by Western blotting. (C) APPL1 is modified with K63-linked ubiquitin chains. HEK293 cells were transiently transfected with the untagged APPL1 and the indicated HA-ubiquitin (Ha-Ub) constructs. After 48 h the cells were treated with 5 μM MG132 for 1 h prior to lysis in RIPA buffer. Lysates and immunoprecipitates with anti-APPL1 antibodies were blotted for HA and APPL1. Asterisks indicate a position of unmodified APPL1. (D) Ubiquitination of APPL1 is enhanced by Nedd4 ubiquitin ligase. HEK293 cells were transiently transfected with the indicated constructs, and 48 h later lysed in RIPA buffer. Lysates and APPL1 immunoprecipitates were resolved by SDS-PAGE and blotted as indicated. Asterisks indicate a position of HA-tagged mouse Nedd4.

We wanted to investigate whether relocalization of APPL1 to ubiquitin-rich aggresomes is related to any changes in its own ubiquitination status. To check if APPL1 is targeted by ubiquitin, we overexpressed APPL1 together with HA-ubiquitin in HEK293 cells. In such overexpression system we detected additional slower-migrating bands recognized by anti-APPL1 antibodies in APPL1 immunoprecipitates (Fig. 5A). One strong and one weak additional bands were visible, each with an apparent size difference of approximately 10 kDa (marked with arrowheads, Fig. 5A). The HA antibody recognizing the tagged ubiquitin detected a high molecular weight smear in APPL1 immunoprecipitates. The additional bands were not present in APPL1 immunoprecipitates from cells lacking the overexpression of ubiquitin. On the other hand, such additional bands of APPL1 were visible in the lysates upon long exposure (Fig. 5A). Thus, APPL1 is subjected to polyubiquitination in ubiquitin-transfected HEK293 cells.

In order to eliminate potential artifacts which could result from protein overexpression, we checked the ubiquitination of endogenous APPL1 in HeLa cells. We failed to detect ubiquitinated APPL1 under control conditions, but we could see polyubiquitinated species of APPL1 upon overnight treatment with MG132 (Fig. 5B). Again, in the APPL1 immunoprecipitates there were multiple bands recognized both by anti-APPL1 and anti-ubiquitin antibodies, as well as a ubiquitin-immunoreactive smear of high molecular weight (Fig. 5B). Thus, endogenous APPL1 is ubiquitinated in HeLa cells under proteasomal stress conditions.

K63-linked polyubiquitination has been reported to promote sequestration of misfolded proteins into aggresomes and their subsequent clearance by autophagy [36]. We wished to determine if the ubiquitin chains conjugated to APPL1 are linked by K63. Thus we overexpressed APPL1 in HEK293 cells together with the wild type HA-tagged ubiquitin or one of the two mutants: with all lysines but K63 mutated to arginine (K63-only), or with K63 single lysine-to-arginine replacement (K63R). The extent of modifications of overall cellular proteins by both ubiquitin mutants was lower than in the case of wild type ubiquitin, which was visible in the lysates probed with anti-HA antibody (Fig. 5C). An anti-HA-reactive band above the size of APPL1 appeared in immunoprecipitates of APPL1 from cells expressing wild type and K63-only ubiquitin (Fig. 5C). In addition, an HA-immunoreactive smear of high molecular weight was formed in the APPL1 immunoprecipitates by the wild type and K63-only ubiquitin, but not by the K63R mutant (Fig. 5C). This result indicates that the ubiquitin molecules conjugated to APPL1 can be linked via a K63-mediated bond.

One of E3 ubiquitin ligases participating in the modification of endocytic targets via K63-linked ubiquitin chains is Nedd4 (neural precursor cell-expressed developmentally downregulated gene 4) [37]. We overexpressed APPL1 and FLAG-ubiquitin with or without human untagged or mouse HA-tagged Nedd4 in HEK293 cells. Compared to control cells, we could detect much stronger, multiple APPL1-immunoreactive bands both in the lysates and the APPL1 immunoprecipitates from cells transfected with the Nedd4 ligases (Fig. 5D). In contrast, overexpression of RING-type E3 ligase c-Cbl did not enhance the amount of ubiquitinated APPL1 despite increasing overall ubiquitination of cellular proteins. This would suggest that Nedd4 is a specific E3 ubiquitin ligase for APPL1.

### Solubility of APPL1

**Fig. 6:**
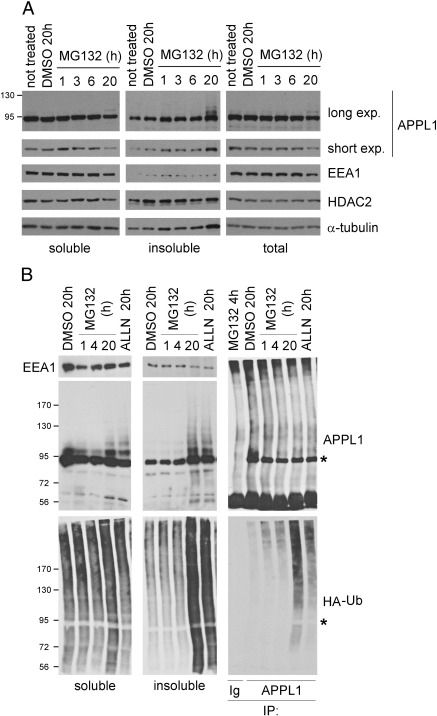
Solubility of APPL1. (A) Prolonged administration of MG132 increases insolubility of APPL1. HeLa cells were treated with DMSO or 10 μM MG132 for the indicated times. Cells were either lysed in hot sample buffer (for total cell lysates) or in RIPA buffer, in which case the detergent-soluble and -insoluble fractions were collected. Equal amounts of protein were resolved by SDS-PAGE and blotted for APPL1 (upper and lower panels depict long and short exposure times, respectively), EEA1, HDAC2 and α-tubulin. (B) HEK293 cells transfected with APPL1 and HA-ubiquitin (HA-Ub) were treated as indicated, lysed in RIPA buffer and extracted into the detergent-soluble and -insoluble fractions which were blotted for EEA1, APPL1 and HA. Immunoprecipitates of APPL1 and control rabbit immunoglobulins (Ig) from the soluble cell fraction were blotted for APPL1 and HA. Asterisks indicate a position of unmodified APPL1.

As aggresomes contain high amounts of detergent-insoluble proteins [19], we hypothesized that the localization of APPL1 to these structures could result from its enhanced insolubility. To assess if the treatment with MG132 alters the solubility of APPL1, we examined the total, soluble and insoluble fractions of HeLa cells treated with DMSO or MG132 from 1 to 20 h. Upon protein extraction with RIPA buffer, APPL1 was almost completely soluble in untreated and DMSO-treated cells. Its solubility was decreased with the increasing time of MG132 treatment (Fig. 6A, APPL1 short exposure). Accordingly, we saw a corresponding increase of APPL1 levels in the detergent-insoluble fraction. Upon 20-h treatment with MG132, additional APPL1-immunoreactive bands were detected, in particular in the insoluble cell fraction (Fig. 6A, APPL1 long exposure). As controls we probed for EEA1 (an endosomal protein which remains soluble after detergent extraction), HDAC2 (a nuclear, chromatin-bound and predominantly insoluble protein) and α-tubulin. While the solubility of EEA1 was not altered, HDAC2 and α-tubulin displayed a slight decrease in solubility upon MG132 treatment (Fig. 6A). With respect to the total protein levels, 20 h of MG132 treatment induced decrease in APPL1 and EEA1, but not the other tested proteins (Fig. 6A). This data suggested that APPL1 becomes insoluble under the same conditions that evoke APPL1 clustering.

**Fig. S5:**
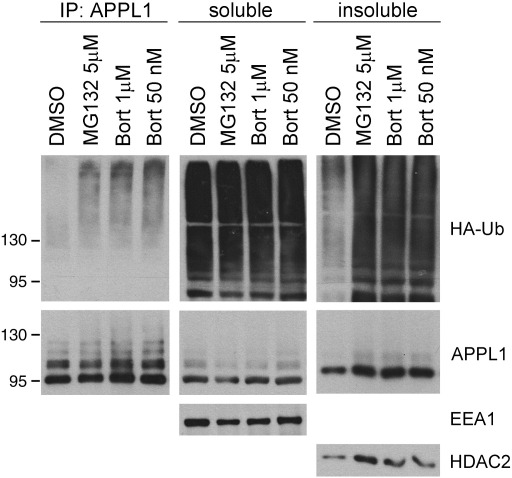
Effects of bortezomib on APPL1 ubiquitination and insolubility. HEK293 cells transiently transfected with APPL1 and HA-ubiquitin (HA-Ub) were treated with indicated inhibitors for 20 h, lysed and processed as in Fig. 6B.

In order to check whether ubiquitin conjugates could correspond to the observed additional bands of APPL1 appearing in an insoluble fraction of HeLa upon prolonged MG132 treatment (Fig. 6A), we performed a similar experiment in HEK293 cells transiently transfected with APPL1 and HA-ubiquitin. We blotted the soluble and insoluble fractions for HA, APPL1 and EEA1, and performed an immunoisolation of APPL1 from the soluble fraction (Fig. 6B). While MG132 only moderately increased the overall level of the soluble ubiquitinated proteins upon 20-h treatment, much more ubiquitinated proteins accumulated in an insoluble cell fraction upon 20-h treatment by MG132 or ALLN (Fig. 6B). In agreement with the data from HeLa cells, both these treatments led to enhanced insolubility of APPL1 and the appearance of multiple APPL1 bands in an insoluble fraction of HEK293 cells (Fig. 6B). In addition, the prolonged MG132 or ALLN treatment caused an increase in the levels of ubiquitinated species present in APPL1 immunoprecipitates (Fig. 6B). Similar results were obtained using two concentrations of bortezomib, that after 20 h caused increased ubiquitination and insolubility of APPL1 (Fig. S5). Thus, proteasomal stress induces ubiquitination of APPL1 and the formation of clusters of ubiquitinated insoluble APPL1.

### Changes in APPL1 distribution and solubility depend on protein synthesis

**Fig. 7:**
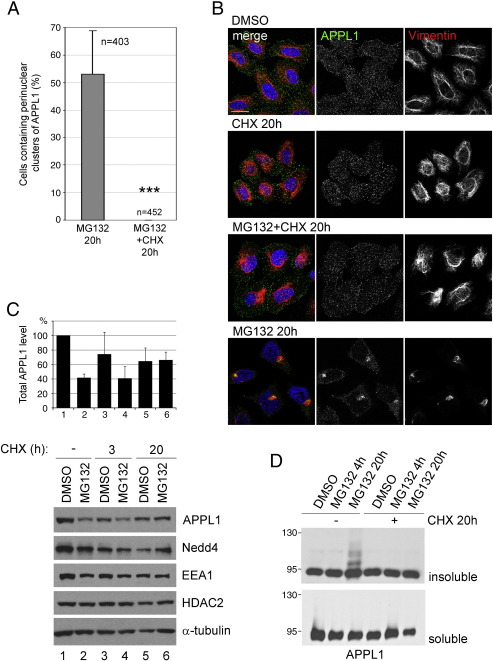
Clustering of APPL1 upon MG132 administration depends on protein synthesis. (A) Quantification of the perinuclear clusters of APPL1. HeLa cells were seeded on coverslips and treated with 5 μM MG132 for 20 h with or without 10 μg/ml cycloheximide (CHX). Six random images taken with a maximally opened confocal pinhole were analyzed for each condition; the cells containing clustered APPL1 were counted and expressed as a percentage of all scored cells (n indicates the number of scored cells). The graph shows an average of two independent experiments with standard deviation. ***, *p* < 0.001 compared with control MG132 alone (Mann–Whitney test). (B) HeLa cells were treated for 20 h with control DMSO, 10 μg/ml cycloheximide, 10 μM MG132 alone or both together. The cells were fixed and stained for APPL1, vimentin and DAPI (blue). Bar, 20 μm. (C) HeLa cells were treated with DMSO or 10 μM MG132 with or without 10 μg/ml cycloheximide for the indicated times prior to lysis in hot sample buffer. Equal protein amounts were resolved on SDS-PAGE followed by detection of levels of the indicated proteins by Western blot. The graph shows quantification of APPL1 total protein levels from Western blot bands, with average values and standard deviation obtained from two separate experiments. Calculation has been performed using ImageJ software. (D) HeLa cells were treated as indicated, lysed in RIPA buffer, centrifuged and extracted into the soluble and insoluble fractions that were subjected to Western blotting using anti-APPL1 antibodies.

Efficient protein biosynthesis is prerequisite for the formation of aggresomes stimulated by bortezomib [38]. We checked how the inhibition of translation with cycloheximide affects the changes in APPL1 distribution and insolubility, and in particular the formation of clusters. In addition to MG132, we incubated HeLa cells with 10 μg/ml cycloheximide to block protein synthesis. We observed a complete loss of clusters in the cells co-treated with MG132 and cycloheximide for 20 h, in comparison to over 50% of cells containing clusters under MG132-only condition (Fig. 7A). As expected, co-staining of APPL1 and vimentin showed lack of aggresomes in the cells co-treated with MG132 and cycloheximide for 20 h, like in control cells and in clear contrast to the cells treated with MG132 alone (Fig. 7B).

Since impairment of proteasome machinery results in an accumulation of proteasomally degraded proteins, we checked for total levels of APPL1 in cells treated with MG132 with or without cycloheximide. HeLa cells treated for 20 h with DMSO or MG132 were incubated for the last 3 h or for the whole 20 h with cycloheximide. The normalized amounts of lysates were blotted for APPL1, Nedd4, EEA1, and control proteins: HDAC2 and α-tubulin (Fig. 7C). Cycloheximide decreased the APPL1 level after 3 and 20 h (compare lanes 1, 3, and 5, Fig. 7C). In agreement with data from Fig. 6A, 20 h of MG132 treatment decreased the amount of APPL1 (compare lanes 1 and 2, Fig. 7C). Interestingly, co-treatment with cycloheximide resulted in stabilization of the total APPL1 level (compare lanes 5–6 with 1–2, Fig. 7C). In parallel, blocking of protein synthesis decreased the levels of Nedd4 and EEA1. In fact, the levels of EEA1 were affected by cycloheximide and MG132 treatment in a manner similar to that described for APPL1 (Fig. 7C). On the other hand, the levels of control proteins, HDAC2 and α-tubulin, were decreased by 20-h treatment with cycloheximide, and not affected by MG132 co-treatment (Fig. 7C). We also blotted for APPL1 in the soluble and insoluble HeLa fractions after co-treatment with MG132 and cycloheximide. Strikingly, the ladder of APPL1 appearing upon 20-h MG132 treatment was not present in the cells co-treated with cycloheximide (Fig. 7D). This suggests that the impairment in protein synthesis results in a coordinated lack of aggresomes and APPL1 clusters, which is accompanied by a lack of accumulation of ubiquitinated insoluble APPL1.

## Discussion

Recent years brought a number of reports that describe the participation of an adaptor protein APPL1 in processes related to cellular growth, proliferation and survival, which at the molecular level are linked with signal transduction, membrane traffic, and gene transcription [14]. The ubiquitin–proteasome system is closely related to all the above processes, and its dysfunction is associated with a number of pathologies. When the proteasome function is compromised, or upon overexpression of proteins normally degraded in proteasomes, ubiquitinated, misfolded and insoluble proteins accumulate and become sequestered into large perinuclear inclusion bodies called aggresomes [39].

Given the growing importance of APPL endosomes, we wished to characterize their fate upon proteasomal stress. Strikingly, we observed a solubilization of APPL1 from endosomal membranes which made further observations of this endosome class impossible, since APPL1 is its unique marker. Instead, a prolonged treatment with all tested proteasome inhibitors caused clustering of APPL1 protein in the perinuclear region. Employing the commonly accepted criteria, we confirmed that endogenous APPL1 is recruited to aggresomes formed upon proteasomal inhibition. This discovery is particularly significant as there are only a handful of known endogenous proteins that become sequestered in aggresomes.

It could be expected that the formation of an aggresome interferes with the proper localization of perinuclear cellular structures, such as the Golgi complex, mitochondria and lysosomes, which was confirmed in our observations. However, APPL1 endosomes are preferentially located at the cell periphery, and proteasome inhibition seems to affect specifically their marker protein APPL1 but not a marker of the canonical early endosomes EEA1. MG132 markedly decreases APPL1 labeling of endosomes, while the total amount of APPL1 protein in the cell is only slightly changed. The perinuclear APPL1 clusters form in about 50% of cells treated with MG132 for 20 h, and they meet the criteria describing aggresome formation: (a) the perinuclear clusters of APPL1 are surrounded by a cage of vimentin (Fig. 3A); (b) the APPL1 and ubiquitin clusters co-localize in the same focal plane of an optical section (Fig. 3B); (c) clustering of APPL1 is inhibited by a microtubule-disrupting agent nocodazole, which also prevents aggresome assembly [19] (Fig. 3C); (d) upon MG132 treatment APPL1 is ubiquitinated (Fig. 5B); (e) upon proteasome inhibition APPL1 accumulates in a detergent-insoluble fraction (Fig. 6); (f) clustering and ubiquitination of APPL1 depend on protein synthesis (Fig. 7). Based on this evidence, we conclude that proteasomal inhibition induces recruitment of ubiquitinated and aggregated APPL1 to aggresomes. In HEK293 cells overexpressing ubiquitin, APPL1 becomes polyubiquitinated with a contribution of K63-linked ubiquitin chains and the ubiquitin ligase Nedd4 can enhance such polyubiquitination reaction (Fig. 5). This is particularly interesting in the view of recent reports showing that the non-degradative K63-linked polyubiquitination promotes sequestration of proteins into aggresomes [36,40].

There could be at least three explanations for the observed recruitment of APPL1 to aggresomes induced by the proteasomal stress. Firstly, if APPL1 was normally a target for proteasomal degradation, the blockage of proteasomes might have caused an excessive accumulation and aggregation of APPL1. Since we do not observe an increase in the total APPL1 protein level upon MG132 treatment, this simple explanation is unlikely. Secondly, proteasomal stress could cause damage to APPL1 which stays misfolded and aggregated due to the sequestration of cellular chaperones that could otherwise help to refold it. Thirdly, APPL1 might co-assembly with other aggregation-prone proteins. Since APPL1 co-localizes with aggregates formed by overexpressed GFP-Eps15 upon MG132 treatment (Fig. 3D), one such candidate could be ubiquilin, which has both ubiquitin-binding and ubiquitin-like domains, that binds to Eps15 and recruits it to aggresomes [34,35].

It is currently believed that aggresomes represent a protective cellular response to stress conditions. Importantly, the ubiquitin–proteasome system and protein aggregation are implicated in the pathogenesis of several genetic and neurodegenerative disorders [41]. The proteasome inhibitor bortezomib used in our study is approved for treatment of patients with relapsed and refractory multiple myeloma, and its antineoplastic activity has been reported in a variety of solid and hematological cancers [42]. A new generation of proteasome inhibitors with improved pharmacological properties are being developed and await testing for their efficiency in treatment of other types of malignancies and immune-mediated disorders [43,44]. Our studies suggest that in clinical practice such proteasome inhibitors may have some impact on endocytic adaptor proteins, as they can affect the localization, ubiquitination and solubility of APPL1. An identification of the signaling pathways that promote aggresome formation and clearance could be vital for elucidating the pathophysiology of many diseases associated with protein misfolding. It would be interesting to check if APPL1 co-localizes with aggresomes formed under disease-relevant conditions other than proteasomal inhibition, such as overexpression of huntingtin with polyglutamine expansion or the deletion mutant of the cystic fibrosis transmembrane regulator (CFTR).

The following are the supplementary materials related to this article.

Suppl. Table 1 The results of morphometric image analysis of APPL1- and EEA1-positive endosomes performed by MotionTracking. The table shows the raw data and the values calculated as a percentage of control values obtained from DMSO-treated cells. AU, arbitrary units; SD, standard deviation.

## Authors’ contribution

I.P. designed and performed the experiments and wrote the paper, L.S. analyzed the microscopical data with MotionTracking, Y.K. constructed the MotionTracking software and provided training of its usage, and M.M. provided conceptual advice and support and edited the manuscript.
